# Effects of a Furrow-Bed Seeding System on Stand Establishment, Soil Bacterial Diversity, and the Yield and Quality of Alfalfa Under Saline Condition

**DOI:** 10.3389/fpls.2022.919912

**Published:** 2022-06-09

**Authors:** Juanjuan Sun, Jinmei Zhao, Tengwei Zhang, Linqing Yu, Ke Jin

**Affiliations:** ^1^Institute of Grassland Research of Chinese Academy of Agricultural Sciences, Hohhot, China; ^2^Inner Mongolia Academy of Grassland Science, Hohhot, China

**Keywords:** *Medicago sativa* L., furrow-bed seeding, soil bacterial diversity, Na^+^ concentration, yield, soil moisture

## Abstract

Salt stress account for large decreases in crop yield all over the world. Furrow-bed system is an efficient practice to promote plant growth in saline soil. However, the effects of Furrow-bed system on the soil environment and the growth of alfalfa (*Medicago sativa* L.) in salinity are not clear. For a wider and more detail evaluation, alfalfa were planted in saline sandy loam soil in fall, the effects of two plant systems (FU, furrow-bed seeding system; FL, flat-bed seeding system) on soil moisture, root zone salinity, soil microbial community structure, seedling emergence number in the early stage of the growth period and soil nutrient contents, alfalfa production characteristics in the second growth year were determined in a 2-year field experiment. The result showed that, compared with FL, FU resulted in increased soil moisture content and seedling emergence, and significantly reduced relative abundance of Actinobacteria and Choroflexi in soil, but it did not affect root zone salinity at the seedling stage. In April of second growth year, the soil salinity was lower, and the soil available phosphorus, potassium, nitrogen, and soil organic matter contents of the root zone were higher in FU than in FL. Compared with FL, FU resulted in increased yield (by 37.5%), protein content (by 3.6%), and potassium concentration (by 33.2%), and decreased ash content (by 7.7%), and sodium concentration (by 19.0%) in alfalfa plants. Pearson’s correlation analysis indicated that the increased yield was positively correlated with seedling emergence, soil available potassium, total nitrogen, and organic matter contents, and shoot potassium content and negatively correlated with shoot sodium content. The relative abundance of Actinobacteria was negatively correlated with alfalfa ash, calcium, and sodium concentrations, and positively correlated with shoot potassium content. Taken together, the results indicate that Furrow-bed seeding in early fall alleviated salt stress of alfalfa and have the potential to enhance the yield and quality of alfalfa cultivated in saline soils by improving the soil environment and regulating the growth and physiology of alfalfa.

**Graphical Abstract:**
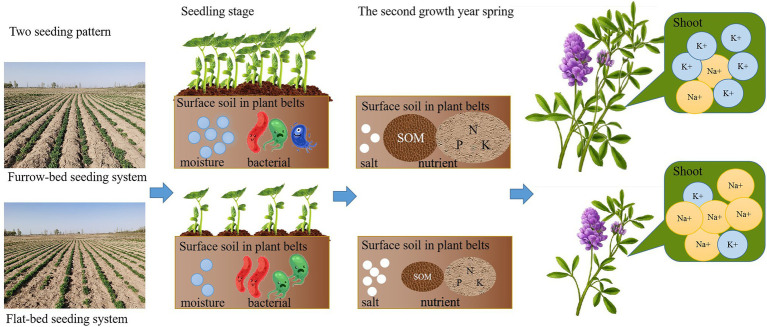

## Highlights

1. Furrow-bed seeding system increased soil moisture content and alfalfa seedling emergence number in the seedling stage.
2. Seeding pattern derived soil microbial community change in seedling stage in saline soil.
3. Furrow-bed seeding system decreased root zone salinity in spring.
4. Alfalfa shoot Na^+^ concentration was decreased and yield was enhanced by furrow-bed seeding system.

## Introduction

Soil salinization is a growing problem for agriculture worldwide (Deinlein et al., 2014), more than 6% of the world’s total land area are salt affected, especially in arid and semiarid regions (Munns and Tester, 2008). Gansu, Inner Mongolia, Xinjiang, Ningxia, Heilongjiang, and Hebei provinces are the major alfalfa-growing areas in China. Although these six provinces produce 89.9% of Chinese high-quality alfalfa, they have almost two-thirds (65.7%) of the area of saline soil in China (Fan et al., 2001). Soil salinity, limited rainfall in spring, high evapotranspiration rates, and poor water management are among the main challenges for agricultural production in this area.

Alfalfa is the most important forage crop and extensively cultivated in the world, and large alfalfa-growing area are known to be subjected to salt stress (Anower et al., 2013). Although it is classified as moderately salt tolerant (Noble et al., 1984), it has been well documented that salt stress inhibits alfalfa shoot growth and increases the shoot sodium (Na^+^) concentration (Sun et al., 2016). Salinity negatively affects alfalfa growth when the electrical conductivity of soil is above 2–3.5 dSm^−1^, and its effects also vary depending on the stage plant growth and development. Alfalfa is very sensitive to salt stress during germination (Allen et al., 1985), at the seedling stage (Ashraf et al., 1987; Esechie et al., 2002), and at the pre-flowering stage (Noble et al., 1984). The accumulated salt in the soil inhibits seed germination, seedling emergence, and plant growth and development through osmotic effects, by causing nutritional imbalances, or by toxicity of salt ions (Na^+^ and Cl^−^). This leads to sparse germination, stunted plants, and/or reduced crop yield and quality (Latrach et al., 2014). Low soil moisture also negatively affects alfalfa seedling emergence and crop establishment. In fact, soil moisture is one of the most important factors affecting crop productivity (Liu et al., 2010a).

Planting patterns affect water and salt transport by controlling evaporation and distributing rainfall, and they are inexpensive and do not pollute the soil. Thus, the use of suitable planting patterns is an environmentally friendly way to moderate soil salinity (Devkota et al., 2015). In arid and semiarid areas, the soils are highly saline with a low moisture content. Consequently, crop productivity in such areas is low, and it cannot meet local food demands. Controlling salinity in the root zone so that it is below harmful levels (reducing root zone salinity) is one beneficial strategy to improve crop emergence and stand establishment in saline fields (Meiri and Plaut, 1985). Previous studies have shown that a furrow-bed seeding system (FU) can efficiently collect rainfall and leach salt from the root zone (Dong et al., 2009; Sun et al., 2017a), so it may be a promising planting pattern for agricultural reclamation in saline–alkaline areas around the world. Dong et al. (2010) found that over-irrigation before planting in an FU system consisting of alternate parallel ridges and furrows on flat land after leaching improved stand establishment and yield (Dong et al., 2010). This was because salts moved upward by capillary action under an evaporation gradient, and accumulated on the soil surface after planting (Dong et al., 2009). The FU system results in the unequal distribution of salts in the surface soil layers (Meiri and Plaut, 1985). Unequal salt distribution under controlled conditions has been shown to affect the physiological characteristics of alfalfa and improve its growth (Sun et al., 2016, 2017b; Xiong et al., 2020). However, it is unknown whether the use of an FU system can improve the growth and yield of alfalfa and the properties of soil in saline soil environments.

The plastic-covered ridge and furrow rainwater harvesting system (PRRFHS) is a well-known soil–water conservation practice used in crop production (Liu et al., 2014; Cuello et al., 2015), and it is one of the most efficient technical applications for maximizing rainfall use. The PRRFHS can improve soil moisture availability in the crop root zone by collecting water from light rain, and this can significantly increase crop yield and water-use efficiency (Liu et al., 2014). It also reduces evaporation and promotes rainfall infiltration and has been widely used in maize and cotton production (Li et al., 2000). However, mulching with plastic also has some negative effects, including plastic waste, greenhouse gas emissions associated with the production of the film (Cuello et al., 2015), and increased planting costs. To date, there have been no studies on the effects of growing alfalfa in a ridge-furrow crop system with no mulch and with a narrow planting belt under saline conditions.

In the soil microbial community, bacteria are one of the richest and most diverse microbial groups, and they play important roles in many soil processes. They participate in soil nutrient cycling, the decomposition of organic matter and waste, the degradation of pesticides and contaminants, and soil aggregation and humus formation. In addition, they affect the soil structure and the growth and health of plants (Jurburg et al., 2018). Soil microbial populations and community composition are affected by a range of edaphic factors, such as soil physicochemical properties (Wakelin et al., 2007) and soil management practices (Chen et al., 2014). Nutrient substrates (Ling et al., 2017), soil pH (Bainard et al., 2016), moisture (Battin et al., 2003; Nguyen et al., 2018; Yang et al., 2019; Chi et al., 2021), and plant community cover (Bainard et al., 2016) are the main ecological drivers of soil bacterial abundance, diversity, and community composition. Soil carbon (C) and nitrogen (N) contents strongly affect soil bacteria, because they decompose soil organic C and N to obtain energy (Yang et al., 2018). For example, Chloroflexi, Nitrospirae, and Planctomycetes tend to be abundant in nutrient-poor soil where they show slow growth rates and use recalcitrant C substrates (Nie et al., 2018). In contrast, Proteobacteria and Bacteroidetes favor nutrient-rich conditions and utilize labile C materials (Philippot et al., 2013). In addition, soil salinity is a critical factor that influence soil microbial community structure and diversity (Guo et al., 2018; Zhao et al., 2020; Cheng et al., 2021; Li et al., 2021). A previous study found that soil salinity negatively affects the abundance of most bacterial groups, but has little effect on bacterial diversity (Gao et al., 2015). Consequently, determining the effects of different planting systems on the soil bacterial community can shed light on how such planting systems affect plant growth.

Soil ridging with plastic film has been shown to improve rainwater utilization and improve alfalfa yield in semiarid areas where alfalfa production is largely rainfed (Li et al., 2007; Gu et al., 2018), However the width of bare soil between planting belts is increased from 30 to 60 cm (Li et al., 2007), the wider planting belts cause higher evaporation. Numerous field studies have explored the effects of the FU system on soil properties, especially soil moisture content, and plant yield. However, few have explored the effects of the narrow plant row FU system on alfalfa grown in saline soils. In addition, the effects of FU on soil bacterial diversity at the seedling stage and the relationships among soil microbes, soil properties, crop yield, and crop quality need further study. We hypothesize that FU system will decrease soil salinity of root zone and improve alfalfa growth in the salinity soil and also have positive impact on the soil microbial and soil properties. Therefore, the objectives of this study were as follows: (1) to evaluate the effects of the FU seeding system with a narrow plant belt on soil properties (especially salinity), soil bacterial diversity at the seedling stage, and alfalfa yield and quality in the second growth year; and (2) investigate the relationships among soil moisture content, soil microbes, soil nutrient contents, soil salinity, and the growth and physiology of alfalfa plants. Therefore, a 2-year field experiment was conducted to explore these topics in detail.

## Materials and Methods

### Experimental Site

**Figure 1:**
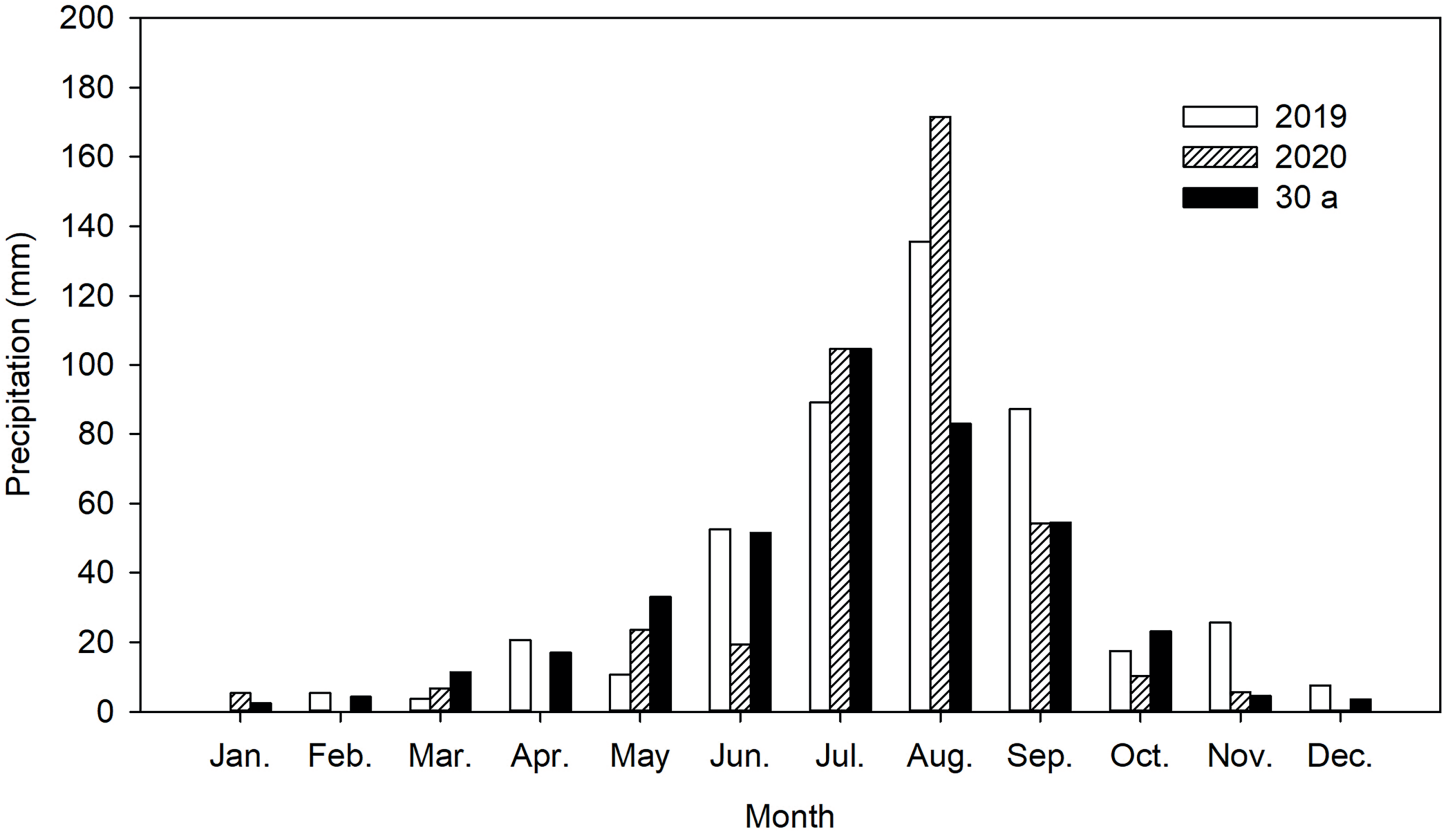
Precipitation distribution from 2019 to 2020 and 30-year average (30 a) at the experimental site.

A field experiment was conducted in an area with saline sandy loam soil in the Hohhot district (111°45′E, 40°36′N) on the Tumochuan plain, China, during two consecutive alfalfa-growing seasons: 2019 and 2020. The site has a typical continental climate with mean annual air temperature of 7.6°C and mean annual maximum and minimum air temperatures of 23.3°C (July) and −11.0°C (January), respectively. The mean annual precipitation is 392.6 mm (average values for 1981–2010), and around 74.8% of the precipitation occurs between July and September. The potential annual evaporation is about 1757.1 mm. Mean annual sunshine exceeds 2829.8 h, and the frost-free period is about 137 days (range, 99 to 183 days). The distribution of precipitation in 2019 and 2020 is shown in Figure 1.

The properties of the background surface soil (0–15 cm) were as follows: 15.9 g kg^−1^ soil organic matter (SOM), 930 mg kg^−1^ total N, 14 mg kg^−1^ available phosphorus (P), and 225 mg kg^−1^ available potassium (K), with an ECe (electrical conductivity of a saturated soil extract) of 15.8 dSm^−1^ in spring and with an ECp (electrical conductivity of pore water) of 1.1dSm^−1^ in early fall. Alfalfa seeds were sown in early August (fall) in 2019.

### Experimental Design and Field Management

**Figure 2:**
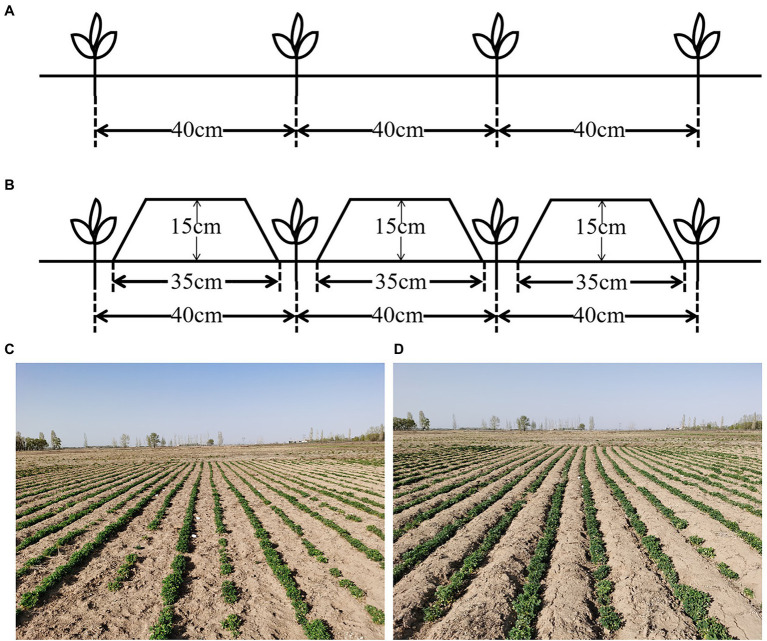
Schematic diagram of **(A)** flat-bed seeding (FL) or **(B)** furrow-bed seeding (FU) systems and the pictures of field experiment in the seedling stage of FL **(C)** or FU **(D)**.

The cold-tolerant alfalfa cultivar *M. sativa* L. cv. “Zhongcao NO.3,” bred by Linqing Yu, Chinese Academy of Agricultural Sciences, was selected for this study, as it is widely cultivated on the Inner Mongolia plateau in China. The experiment comprised two cultivation systems: furrow-bed seeding (FU; cultivation with ridges and furrows) and flat-bed seeding (FL; conventional flat cultivation without ridges). The experiment had a completely randomized block design with four replicates. Each plot was 20.0-m long and 3.2-m wide. Alfalfa seeds were planted on 8th August 2019 using the drill planting method with a hand-pushed vegetable planter. For the FU treatment, the width of the ridges and the furrows was 35 and 5 cm, respectively, and the ridge height was 15 cm. The furrows were leveled as planting belts (Figure 2). The ridge and furrow were made using a tiny furrow machine and the ridges were compacted using a roller. Alfalfa seeds were drill-sown in the furrows in the FU system and then covered with 1 cm soil. All the planting rows in both FU and FL systems had a north–south orientation with 40-cm spacing between adjacent rows. Weeds were controlled manually, with care taken not to destroy the ridge soil crust. Alfalfa plants were harvested manually in 2020.

The alfalfa plants in the plots were not irrigated in 2019 because rainfall was sufficient that year, but were irrigated three times at key stages of growth in 2020. Other field management was conducted according to local agronomic practices.

### Data Collection

Data were collected for rainfall, soil temperature, soil salinity, soil moisture content, seedling emergence number, soil bacterial diversity, soil properties, plant height, plant yield, shoot sodium (Na^+^) concentration, shoot potassium (K^+^) concentration, shoot calcium (Ca^2+^) concentration, and plant nutritional components.

Rainfall at the experimental site was measured using an automatic weather station (WSSTD1, Campbell Scientific, Loughborough, United Kingdom). The number of emerged seedlings (number of alfalfa seedlings at the cotyledon stage) was counted in five 100-cm-long seed row sections and the average number per 100-cm-long seed row in each plot at 4, 6, and 9 days after sowing (DAS) was calculated. The soil moisture content, salinity, and temperature during the seedling stage at 4, 6, and 9 DAS were determined by analyzing 10 surface (0–10 cm) soil samples with a HH2 Moisture Meter and a WET-2 sensor (Delta-T Devices Ltd. Cambridge, United Kingdom; Kargas et al., 2011). For analyses of the soil bacterial community, five surface (0–15 cm) soil samples were collected from random positions along the crop row from each plot in both systems on 9 DAS. The soil samples from each plot were homogenized to form a composite sample and sieved through a 2 mm sieve to remove rocks and roots. A subsample was immediately stored at −80°C until use in molecular analyses.

For determination of the properties of the soil before the experiments started, 10 soil cores were collected from random positions in the experimental field. For determination of soil salinity and the contents of SOM, total N, available P, and available K during the experiment, five soil cores were collected from random positions along the crop row to a depth of 15 cm from both the FU and FL treatment in the spring of 2020 when the alfalfa plants had just turned green. The soil samples from each plot were homogenized to form a composite sample. Subsamples were air-dried, ground, and passed through a 2-mm sieve, then used for analyses of soil salinity, SOM, available K, available P, and total N. Soil was mixed with water (1:5 soil to water ratio) to determine soil salinity. Soil chemical characteristics were tested following the method described by Bao (2018).

### Plant Characteristics

Plant height was measured five times from 17 October 2019 to 3 July 2020. At each measurement time, 20 plants were selected from each plot and the height was measured and the average height of alfalfa were used. In 2020, the yield of the first cut of alfalfa was measured. Alfalfa plants were cut in a 1-m long part of the row, with three replicates per plot. The average yield from plants in 1 m was calculated and used to estimate the yield per hectare. The harvested material was dried at 65°C for 48 h and then weighed to determine dry matter content. Then, the dried samples were ground to pass through 1-mm screen using a laboratory knife mill (FW100, Taisite Instruments, Tianjin, China) for later analysis. Neutral detergent fiber (NDF; Van Soest et al., 1991) and acid detergent fiber (ADF; Robertson and Van Soest, 1981) were measured using an ANKOM fiber analyzer (ANKOM2000; Macedon, NY, United States). Crude ash (ash) content was determined by burning samples in a muffle furnace at 500°C for 5 h and then weighing the residue. Total nitrogen (total N) content was determined by the Kjeldahl procedure (Krishnamoorthy et al., 1982); crude protein (CP) was determined by multiplying the total N by 6.25. Ions were extracted by shaking ground leaf samples in 0.5 M HNO_3_ in vials for 48 h. Then, the diluted extracts were analyzed to determine their Na^+^, K^+^, and Ca^2+^ contents using an M410 flame photometer (Sherwood, Cambridge, United Kingdom).

### DNA Extraction

DNA was extracted from 0.25-g soil using an E.Z.N.A.^®^Soil DNA Kit (Omega Biotek, Norcross, GA, United States) according to the manufacturer’s instructions. The reagents in this kit were designed to isolate DNA from trace amounts of sample, and are effective for isolating DNA from most bacteria. Nucleic acid-free water was used as the blank. The total DNA was eluted in 50-μl elution buffer and stored at −80°C until PCR analyses by the LC-Bio Technology Co., Ltd. (Hang Zhou, Zhejiang Province, China).

### PCR Amplification and 16S rDNA Sequencing

The V3–V4 region of the prokaryotic (bacterial and archaeal) small-subunit 16S rDNA gene was amplified with slightly modified versions of the primers 338F (5′-ACTCCTACGGGAGGCAGCAG-3′) and 806R (5′-GGACTACHVGGGTWTCTAAT-3′). The 5′ ends of the primers were tagged with specific barcodes for each sample and universal sequencing primers (Logue et al., 2016).

Each PCR amplification reaction mixture contained 25-ng template DNA, 12.5-μl PCR premix, 2.5 μl each primer, and PCR-grade water to complete the volume to 25 μl. The thermal cycling conditions to amplify the prokaryotic 16S fragments were as follows: initial denaturation at 98°C for 30 s; 35 cycles of denaturation at 98°C for 10 s, annealing at 54°C/52°C for 30 s, and extension at 72°C for 45 s; and then final extension at 72°C for 10 min. The PCR products were confirmed by 2% agarose gel electrophoresis. Throughout the DNA extraction process, ultrapure water, instead of a sample solution, was used to exclude the possibility of false-positive PCR results as a negative control. The PCR products were purified using AMPure XT beads (Beckman Coulter Genomics, Danvers, MA, United States) and quantified by Qubit (Invitrogen, Carlsbad, CA, United States). The amplicons were prepared for sequencing using a Library Quantification Kit for Illumina (Kapa Biosciences, Woburn, MA, United States), and the size and quantity of the amplicon library were assessed using an Agilent 2100 Bioanalyzer (Agilent, Palo Alto, CA, United States). The PhiX Control library (v3; Illumina) was combined with the amplicon library (expected at 30%). The libraries were sequenced with 300PE MiSeq runs. One library was sequenced with both protocols using standard Illumina sequencing primers, eliminating the need for a third (or fourth) index read. The sequencing data were submitted to the NCBI Sequence Read Archive database (accession number: PRJNA826619).

### Data Analysis

Samples were sequenced on the Illumina MiSeq platform according to the manufacturer’s recommendations (LC-Bio). Paired-end reads were assigned to samples based on their unique barcode and truncated by cutting off the barcode and primer sequences. Paired-end reads were merged using FLASH. Quality filtering of the raw tags was performed under specific filtering conditions to obtain high-quality clean tags according to FastQC (V 0.10.1). Chimeric sequences were filtered using Verseach software (v 2.2.4). Sequences with ≥97% similarity were assigned to each representative sequence using the RDP (Ribosomal Database Project) classifier. Differences in dominant species among different groups were detected and multiple sequence alignments were conducted using PyNAST software, which revealed the phylogenetic relationships among different operational taxonomic units (OTUs). Abundance information for OTUs was normalized using a standard sequence number corresponding to the sample with the least sequences. Alpha diversity was determined by calculating four indices (Chao 1, Shannon’s, Simpson’s, and Observed species) using QIIME (V 1.8.0). Differences in beta diversity (species complexity) among samples were detected by a principle co-ordinates analysis (PCoA) conducted using QIIME (V 1.8.0).

### Statistical Analysis

One-way analysis of variance (ANOVA) was used to evaluate statistical significance of the effects of the two seeding patterns on soil properties, plant properties, relative abundance of dominant bacterial phyla, classes, and genera; bacterial community richness, diversity indices, and OTUs. T-test were conducted to ascertain any significant difference between two seeding patterns at the 0.05 probability level. Proc GlM procedure was used for analysis of variance. Levene’s test were used for homogeneity of variance. All above data analysis was performed using SAS version 8.02 (SAS Institute, Cary, NC, United States). SigmaPlot was used to generate bar graphs. Spearman correlation analysis was performed to detect relationships among soil and plant properties and soil fungal abundance, diversity, and relative abundance of dominant bacteria phyla and classes using the package stats of the software R version 3.6.3. Pearson’s correlation analysis was performed to detect relationships between alfalfa yield and soil and plant properties (*n* = 4) in two crop systems using Systat version 12.0 (Systat Software Inc., Chicago, IL, United States). The relationships between the soil bacterial community composition, at both phylum and class level, with soil and plant properties were analyzed with redundancy analysis (RDA) using the package vegan of the software R version 3.6.3.

## Results

### Soil Moisture Content, Soil Salinity, Soil Temperature, and Seedling Emergence

**Figure 3:**
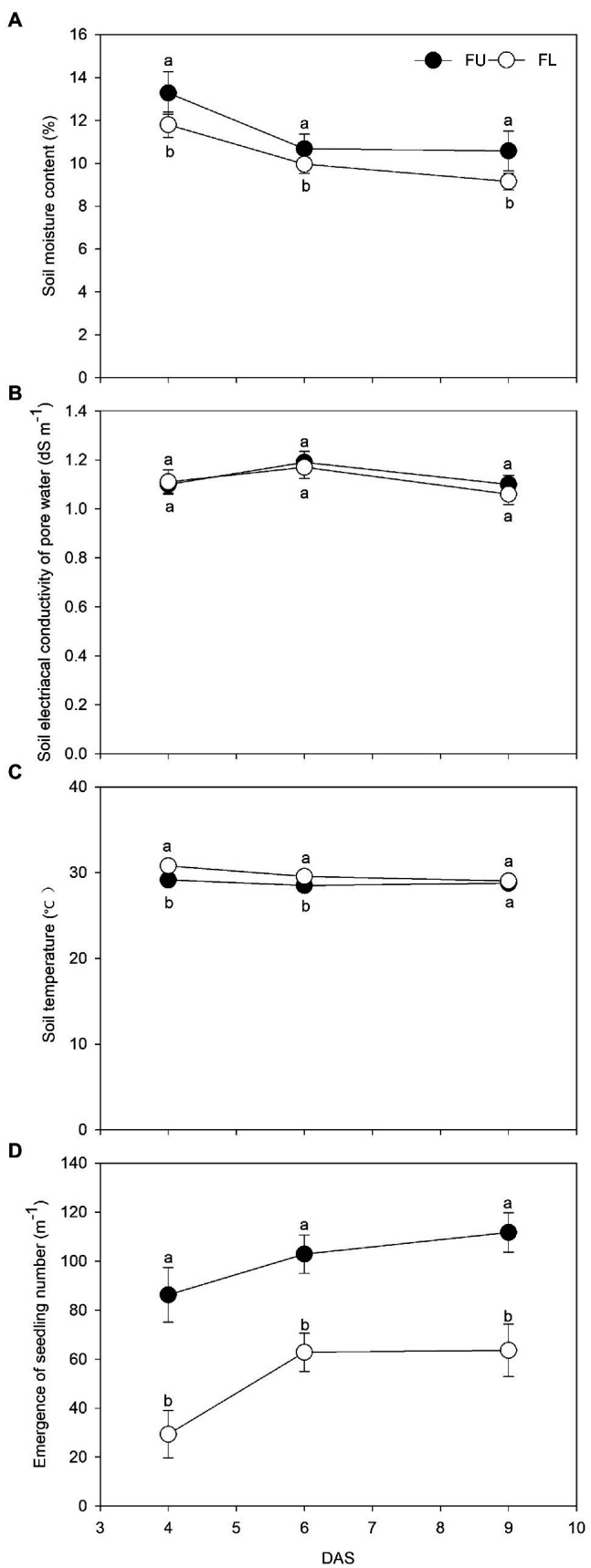
Effects of FU and FL on Soil moisture content **(A)**, soil electrical conductivity of pore water **(B)**, soil temperature **(C)**, and number of emerged seedlings **(D)** of alfalfa 4, 6, and 9 days after sowing (DAS). During the seedling stage. Solid circles show FU treatment; hollow circles show FL treatment. Values are means (*n* = 4) ± SE. Same lowercase capital letters indicate no significant difference between two seeding patterns at *p* ≤ 0.05.

The seeding pattern significantly affected the soil moisture content, soil temperature, and number of emerged seedlings, but not soil EC (Figure 3). At 4, 6, and 9 DAS, compared with the FL system, the FU system increased soil moisture content by 12.5%, 10.2%, and 15.6%, respectively; increased the number of emerged seedlings by 194.1%, 63.8%, and 75.7%, respectively; and decreased the soil temperature by 1.6°C, 1.1°C, and 0.3°C, respectively.

### Inter-Row and Inner-Row Soil Moisture Content During the Vegetative Growth Stage

**Figure 4:**
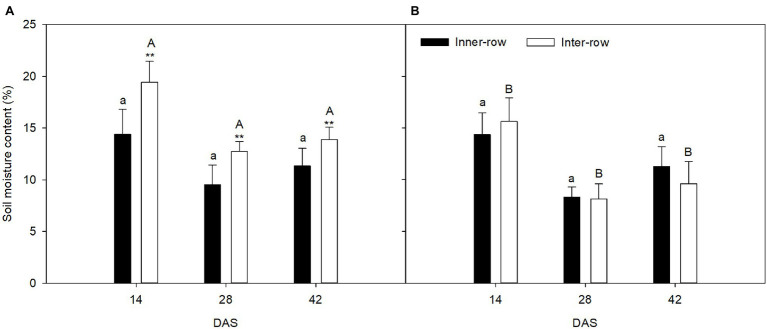
Effects of FU **(A)** and FL **(B)** on soil moisture content of ridge/inter-row and furrow/inner-row at 14, 28, and 42 days after sowing (DAS). Solid bars show furrow/inner-row soil moisture content; hollow bars show ridge/inter-row soil moisture content. Values are means (*n* = 4) ± SE. Asterisks above symbols indicate significant differences in soil moisture content between inter-row and inner-row at *p* ≤ 0.05. Same capital letters indicate no significant difference in inter-row soil moisture content between two seeding patterns at *p* ≤ 0.05. Same lowercase capital letters indicate no significant difference in inner-row soil moisture content between two seeding patterns at *p* ≤ 0.05.

The seeding pattern significantly affected the inter-row soil moisture content, but not the inner-row moisture content (Figure 4). At 14, 28, and 42 DAS, the inter-row soil moisture content was 35.11% higher, 33.44% higher, and 22.37% higher, respectively, than the inner-row soil moisture content in FU. However, the inter-row and inner-row soil moisture contents were not significantly different in FL. The seeding pattern significantly affected the inter-row soil moisture content. At 2, 4, and 6 weeks after sowing, the soil moisture content in FU was 24.38% higher, 56.77% higher, and 44.18% higher, respectively, in FU than in FL.

### Soil Bacterial Alpha Diversity and Beta Diversity Analysis

The seeding pattern had no significant effect on OTU richness, species richness (Chao1 index), and diversity (Shannon’s index). However, the values of OTU richness, Chao1’s, and Shannon’s indices were higher in FU than in FL (Table 1).

**Table 1:** Number of sequences analyzed and observed bacterial community richness and diversity indexes of two seeding patterns obtained for clustering at 97% similarity levels.

Seeding pattern	OTU richness	Observed species	Shannon	Chao 1
FU	6,870 ± 199	6,418 ± 87	11.29 ± 0.07	8,096 ± 115
FL	6,808 ± 29	6,082 ± 126	11.11 ± 0.11	7,681 ± 113

*Values are mean (*n* = 4) ± SE*.

**Figure 5:**
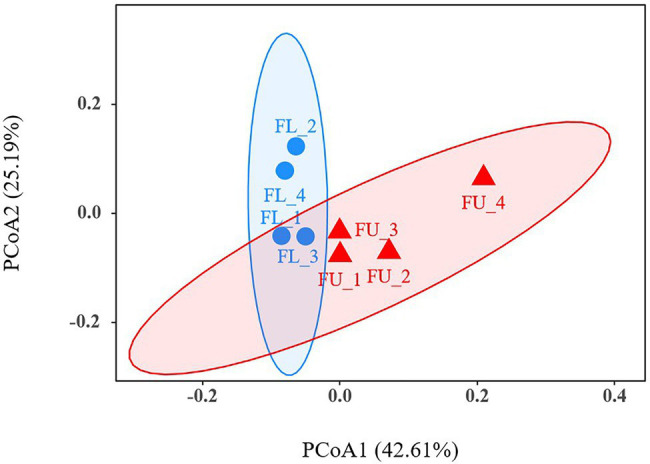
Principal co-ordinates analysis (PCoA) at operational taxonomic unit (OTU) level. The red triangles represent FU soil samples, and the blue circles represent FL soil samples.

A PCoA was used to analyze beta diversity, to identify the differences in soil bacterial community composition between FU and FL (Figure 5). At the OTU level, PCoA analyses showed that the FU soil samples clustered together, and were distinct from FL soil samples, indicating that their bacterial community compositions were dissimilar.

### Taxonomic Composition of Soil Bacterial Communities

**Figure 6:**
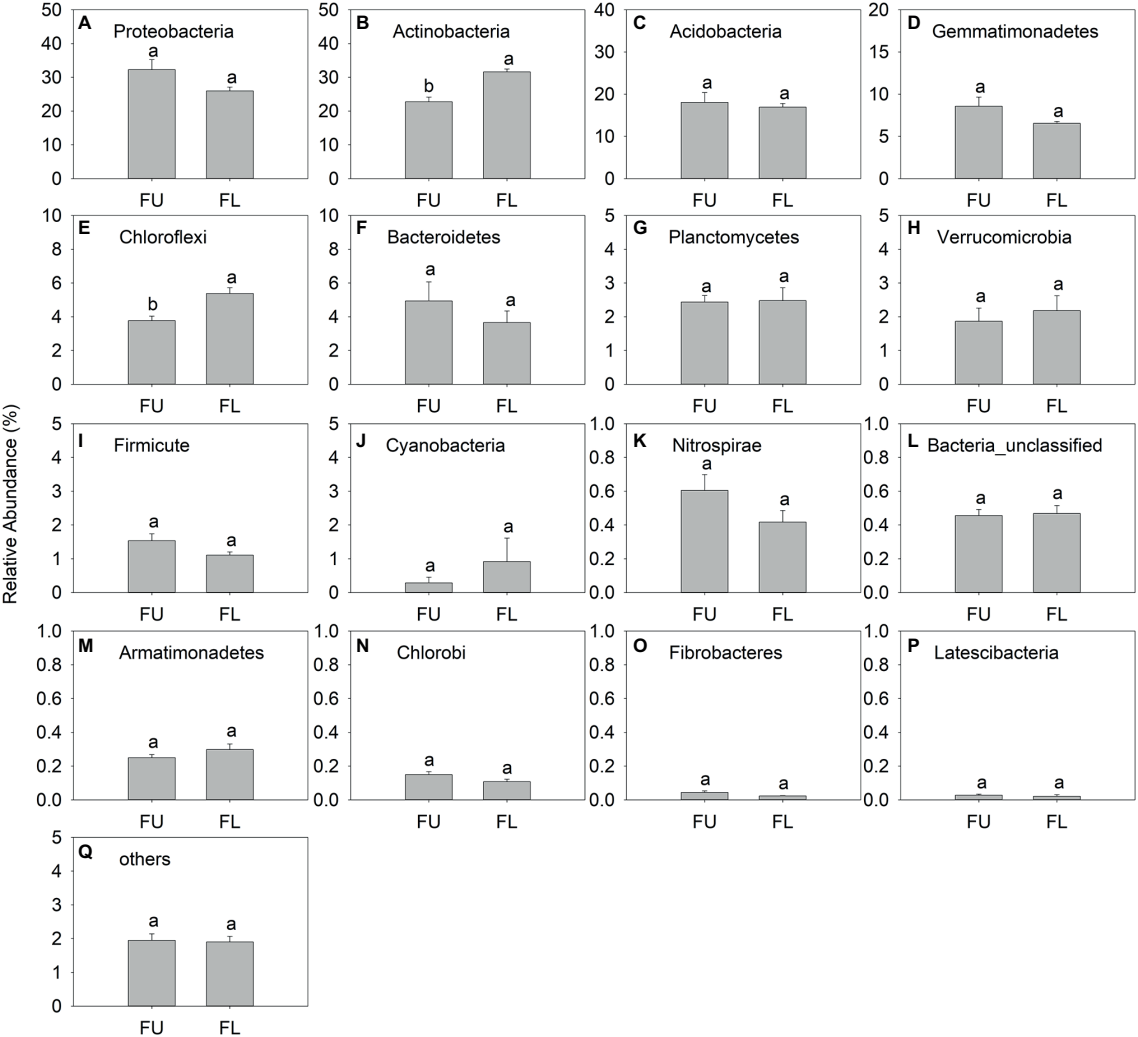
Relative abundance (% of individual taxonomic group) of dominant bacteria phyla (mean ± SE, *n* = 4) in microbial communities following FU and FL of alfalfa. **(A-Q)** represented different soil bacterial at phylum level. Different lowercase capital letters indicate statistically significant difference among communities at *p* = 0.05.

Proteobacteria (relative abundance, 25.97%–32.28%), Actinobacteria (22.76%–31.60%), Acidobacteria (16.92%–18.06%), Gemmatimonadetes (6.54%–8.57%), and Chloroflexi (3.77%–5.38%), were the main bacteria phyla (Figures 6A-E). Latescibacteria was a minor phylum, with relative abundance ranging from 0.021% to 0.028% (Figure 6P). Proteobacteria was the dominant bacterial phylum in FU, but Actinobacteria was the dominant bacterial phylum in FL. The relative abundance of Actinobacteria and Chloroflexi was significantly lower in FU than in FL. The relative abundance of other bacteria groups showed no significant difference between the two seeding patterns (Figure 6).

**Figure 7:**
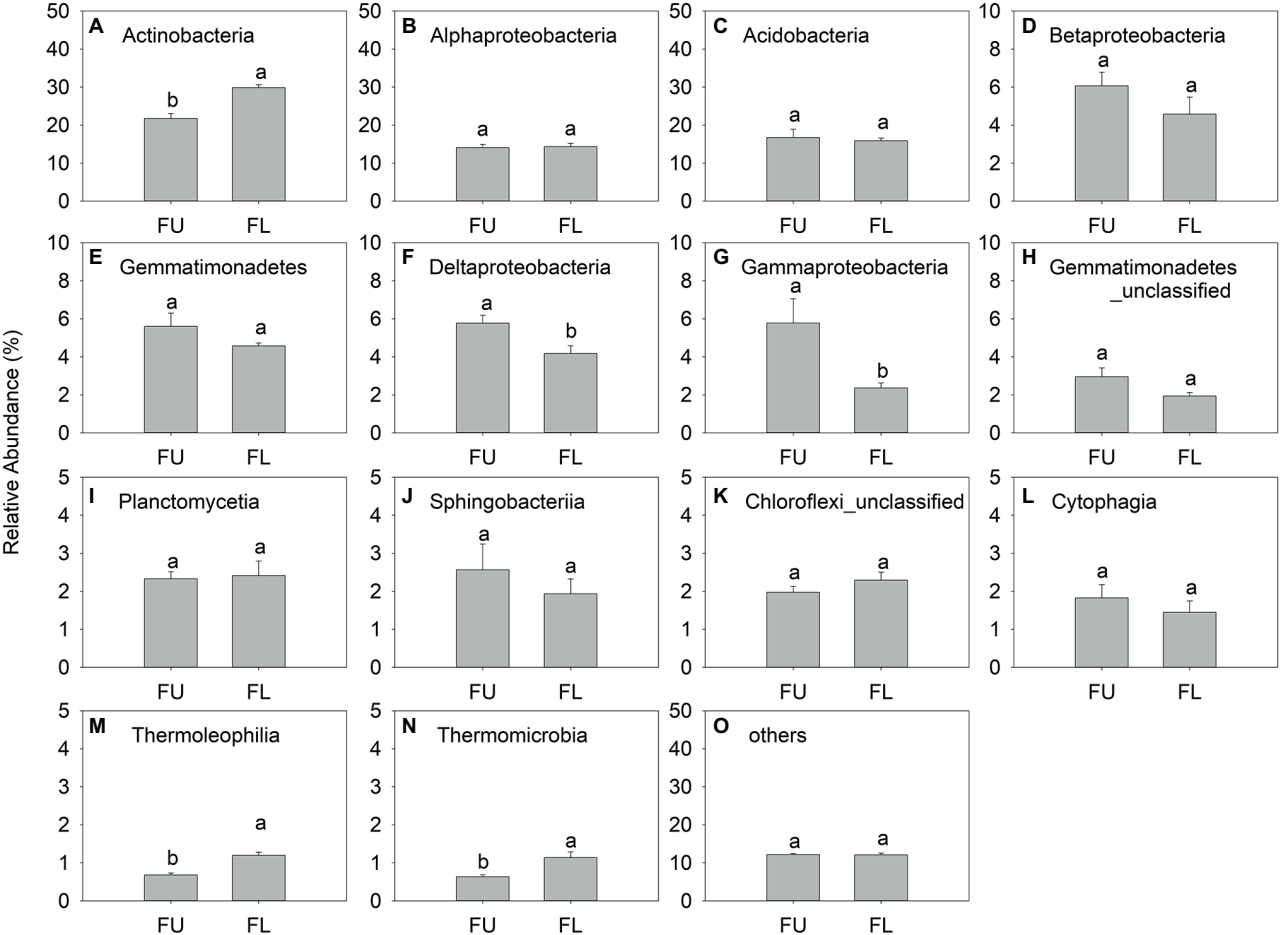
Relative abundance (% of individual taxonomic group) of dominant bacteria classes (mean ± SE, *n* = 4) in microbial communities following FU and FL. **(A-O)** represented different soil bacterial at class level. Different lowercase capital letters indicate statistically significant difference among communities at *p* = 0.05.

The main bacterial classes were Actinobacteria (relative abundance, 21.78%–29.85%), Alphaproteobacteria (14.07%–14.34%), Acidobacteria (15.90%–16.73%), Betaproteobacteria (4.58%–6.06%), Gemmatimonadetes (4.57%–5.59%), and Deltaproteobacteria (4.18%–5.77%; Figures 7A-F). Thermomicrobia was a minor class, with relative abundance ranging from 0.63% to 1.13% (Figure 7N). Actinobacteria was the dominant bacterial class in FU and FL. The relative abundance of Actinobacteria and Thermoleophilia was significantly lower in FU than in FL, while that of Deltaproteobacteria and Gammaproteobacteria was significantly higher in FU than in FL. The relative abundance of other bacterial classes did not differ significantly between the two seeding patterns (Figure 7).

At the genus level, the relative abundance of unclassified Actinobacteria (*Actinobacteria-unclassified*) and unclassified Actinomycetales (*Actinomycetales_unclassified*) were lower in FU soil than in FL soil, while that of unclassified Betaproteobacteria (*Betaproteobacteria_unclassified*) was higher in FU than in FL (Supplementary Figure S1).

### Plant Height at Different Growth Stages

**Figure 8:**
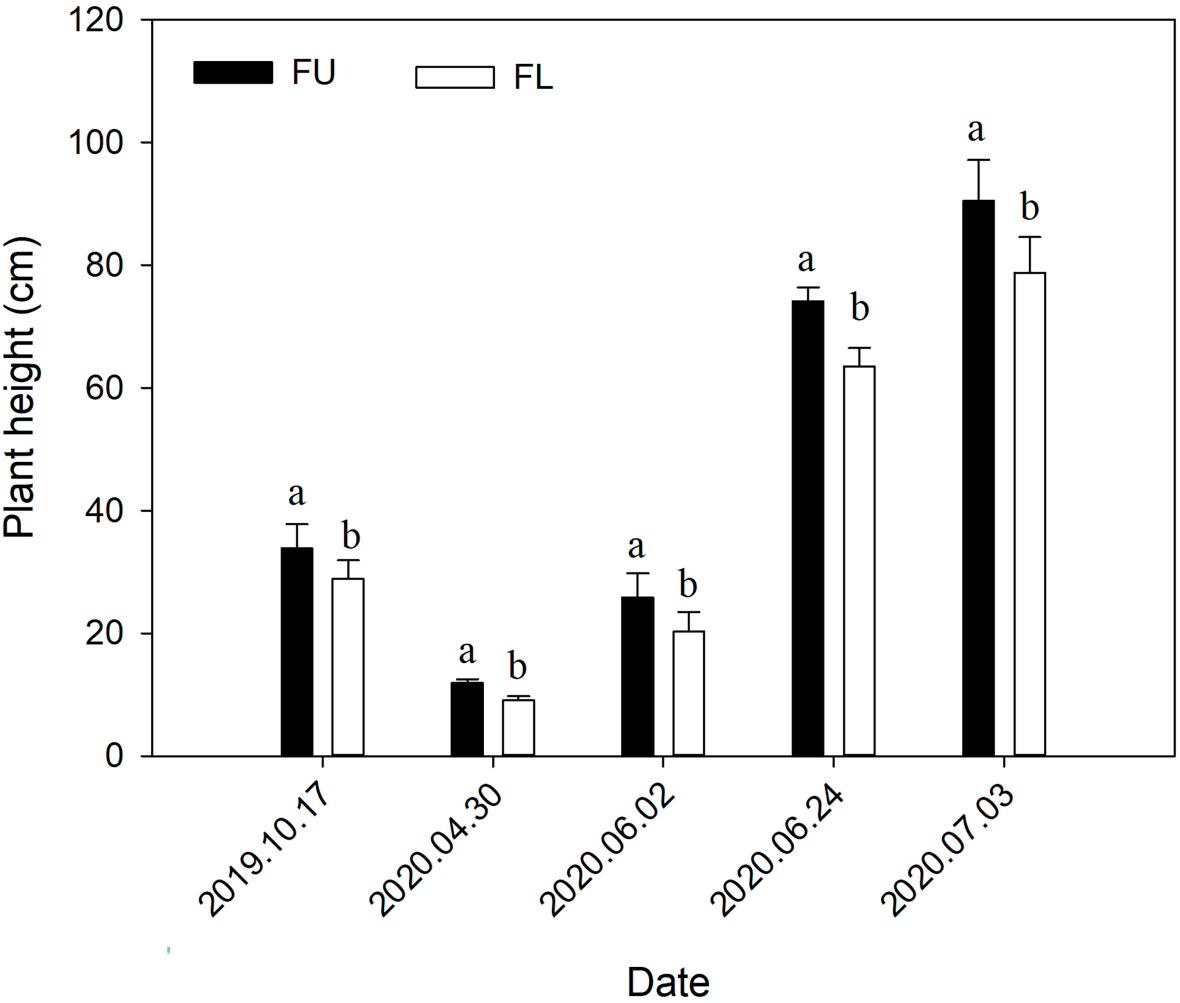
Effects of seeding pattern on alfalfa plant height in 2019 and 2020. Values are means (*n* = 4) ± SE. Different lowercase letters indicate significant difference in plant height between FU and FL at each measurement time (*p* ≤ 0.05).

The seeding pattern significantly affected alfalfa height in both 2019 and 2020 (Figure 8). In 2019, plant height was 17.12% higher in FU than in FL. In 2020, the plant height at the four measurement times was 30.79%, 27.59%, 16.89%, and 14.82% higher in the FU system than in the FL system.

### Soil Properties in April 2020

The soil available K, soil total N, and SOM were significantly higher in FU than in FL, while the ECe was lower in FU than in FL (Table 2). Soil available K, total N, and SOM were 18.3%, 23.8%, and 28.8% higher, respectively, in FU than in FL. The ECe was 25.7% lower in FU than in FL.

**Table 2:** Soil properties (0–15 cm depth) under two seeding pattern treatments in April 2020.

Seeding pattern	Ava_P (mg kg^−1^)	Ava_K (mg kg^−1^)	Total N (g kg^−1^)	ECe (dSm^−1^)	SOM (g kg^−1^)
FU	21.7 ± 0.73a	280.7 ± 8.6a	1.05 ± 0.009a	12.8 ± 0.61b	18.8 ± 0.10a
FL	18.0 ± 3.76a	237.3 ± 5.8b	0.8 ± 0.047b	17.3 ± 1.43a	14.6 ± 0.90b

*Values are mean (*n* = 4) ± SE. ava_P, available P; ava_K, available K; ECe, electrical conductivity of a saturated soil extract; SOM, soil organic matter. Different lowercase letters within each column indicate statistically significant differences at *p* = 0.05*.

### Yield and Nutrient Contents of First-Cut Alfalfa in 2020

The seeding pattern significantly affected alfalfa yield, and the contents of CP, NDF, ADF, ash, and ions in alfalfa plants (Table 3). Compared with FL, FU resulted in increased alfalfa yield, CP content, K^+^ concentration, and Ca^2+^ concentration (by 37.4%, 3.56%, 33.2%, and 17.8%, respectively), and decreased contents of ash and Na^+^ (by 7.71% and 19.01%, respectively).

**Table 3:** Alfalfa characteristics under two seeding pattern treatments at the first cut in 2020.

Seeding pattern	Yield(DM kg/hm^2^)	CP (g kg^−1^ DM)	NDF (g kg^−1^ DM)	ADF (g kg^−1^ DM)	Ash (g kg^−1^ DM)	K^+^ (g kg^−1^ DM)	Ca^2+^ (g kg^−1^ DM)	Na^+^ (g kg^−1^ DM)
FU	7,590 ± 566a	181 ± 1.8a	629 ± 10.9	456 ± 25.4	83.8 ± 2.3b	14.0 ± 0.2a	4.5 ± 0.1a	2.3 ± 0.11b
FL	5,520 ± 405b	174 ± 1.7b	631 ± 26.9	464 ± 21.5	90.8 ± 1.0a	10.5 ± 0.7b	3.8 ± 0.7b	2.8 ± 0.04a

*DM, dry matter; CP, crude protein; NDF, Neutral detergent fiber; ADF, acid detergent fiber. Values are mean (*n* = 4) ± SE. Different lowercase letters within each column indicate statistically significant differences at *p* = 0.05*.

### Relationships Between Soil Bacterial Communities and Soil and Plant Properties

**Figure 9:**
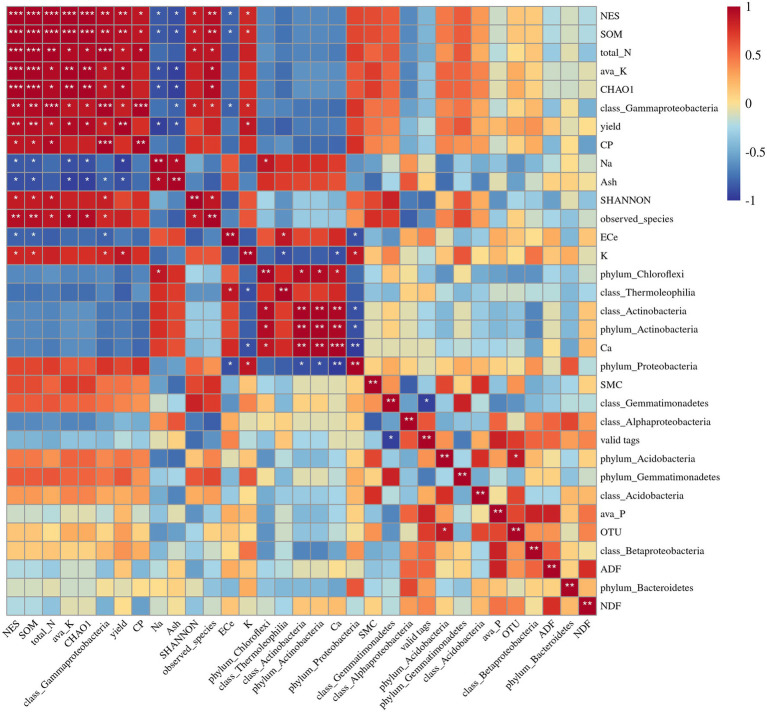
Spearman correlation analysis among soil and plant properties and soil fungal abundance, diversity, and relative abundance of dominant bacteria phyla and classes. NES, number of emergence seedling; SMC, soil moisture content; ava_K, available K; ava_P, available P. The values presented by colors in the heat map correspond to the Spearman correlation coefficient r, which ranged between −1 and 1, where *r* < 0 indicates a negative correlation (blue), *r* > 0 indicates a positive correlation (red), “*” represents *p* < 0.05, “**” represents *p* < 0.01, “***” represents *p* < 0.001.

Spearman correlation analysis indicated that the Observed species index was positively correlated with soil available K, soil total N, SOM, and soil moisture content (Figure 9). The Chao1 species richness index was positively correlated with soil available K, soil total N, and SOM, and negatively correlated with the shoot Na^+^ concentration (Figure 9). The relative abundance of Actinobacteria was highly correlated with alfalfa ash content, shoot Ca^2+^ concentration, and shoot Na^+^ concentration and negatively correlated with shoot K^+^ concentration. The relative abundance of Chloroflexi was positively correlated with the shoot Ca^2+^ and Na^+^ concentrations and negatively correlated with the soil available P concentration. The relative abundance of Deltaproteobacteria was positively correlated with soil total N, SOM, and alfalfa CP content. The relative abundance of Gammaproteobacteria was positively correlated with soil available K, soil total N, SOM, alfalfa CP content, and shoot K^+^ concentration, and negatively correlated with shoot Ca^2+^ and Na^+^ concentrations. The relative abundance of Thermoleophilia was positively correlated with shoot Ca^2+^ concentration and negatively correlated with shoot K^+^ concentration.

### Relationships Between Alfalfa Yield and Soil and Plant Properties in the Second Growth Year

Pearson’s correlation analysis indicated that alfalfa yield was positively correlated with the number of emerged seedlings, and the contents of soil available K, soil total N, and SOM. In addition, alfalfa yield was positively correlated with the shoot K^+^ concentration and negatively correlated with the shoot Na^+^ concentration (Table 4).

**Table 4:** Pearson correlations coefficients between alfalfa yield and soil and plant properties (*n* = 4) under two seeding pattern treatments.

	Ava_P	Ava_K	Total N	ECe	SOM	K^+^	Ca^2+^	Na^+^	NES
Yield	0.239	0.974^**^	0.892^*^	0.106	0.883^*^	0.888^*^	−0.689	−0.891^*^	0.953^**^

*Ava_P, available P; Ava_K, available K; ECe, electrical conductivity of a saturated soil extract; SOM, soil organic matter; NES, number of emergence seedling*.

* *p <* *0.05*,

** *p <* *0.05 (Pearson’s correlation coefficient test)*.

## Discussion

### Effects of FU on Seedling Emergence and Soil Properties at the Seedling Stage

Seedling emergence and establishment are key processes in alfalfa production (Kunzova and Hejcman, 2009). In our study, FU significantly increased the number of emerged seedlings, and this was positively correlated with alfalfa yield (Table 4). Soil moisture is one of the main factors affecting seed emergence and plant growth and development (Silvente et al., 2012; Liao et al., 2021). Even a small change in soil water storage can affect crop productivity (Liu et al., 2010a,b). The higher seedling emergence in the FU system than in the FL system was mainly due to the higher soil moisture content in the FU system. Ridge-furrow cropping is a well-known soil–water conservation practice used in crop production. In our study, the water content in the furrow during the seed germination stage was increased in the FU system, consistent with the results of previous studies (Li et al., 2007; Zhang et al., 2011; Zribi et al., 2015; Liao et al., 2019; Suo et al., 2019). The higher soil moisture content in the furrow was mainly because the soil moisture content was higher in the top 15-cm soil layer (the ridge height was 15 cm) than in the surface soil after the rainy season in early fall. It was not due to rainwater harvesting, because there was no rainfall during the seedling period in our experiment. In our study, the FU system reduced the soil temperature at the seedling stage, because the higher soil moisture in the FU treatment reduced the thermal conductivity compared with that of soil in FL. The lower soil temperature did not inhibit seed germination, because alfalfa seeds begin germinating shortly after planting, when soil temperatures are above 18°C and adequate moisture is present (Summers and Putna, 2008). In our study, FU did not significantly affect the soil salinity at the seedling stage, probably because alfalfa seeds were planted after the rainy season in early fall. At this time, the salt had been leached from the soil by rain, so that the salinity levels in topsoil were low both in FU and FL.

### Effects of FU on Soil Bacterial Diversity

The PCoA plots show that the bacterial community composition was significantly different between FU and FL (Figure 5), indicating that the FU system affected the bacterial abundance and diversity at the seedling stage. As indicated by the higher Chao1 value, the FU system increased the bacterial richness compared with that in the FL system (Table 1). Consistent with this, a previous study found that agricultural management influences the soil microbial community and composition (Chen et al., 2014). In the present study, the higher bacterial richness in the FU system may have resulted from the higher soil moisture content. Previous studies have shown that soil moisture strongly affects soil bacteria (Bainard et al., 2016; Nguyen et al., 2018) and that higher soil moisture content can favor bacterial growth (Nakamura et al., 2003). Interestingly, neither our study nor a previous study (Yang et al., 2019) detected a strong relationship between soil salinity and soil bacterial abundance. This might be because there were only small variations in salinity at the seedling stage, since alfalfa seeds were sown after the rainy season when much of the salt had been leached from the soil.

In the present study, high-throughput sequencing revealed that the dominant bacterial phyla in saline soils were Proteobacteria, Actinobacteria, Acidobacteria, Gemmatimonadetes, and Chloroflexi (Figure 6). These five phyla accounted for an average 85.45% and 86.41% of total bacterial sequences in FU and FL, respectively. This soil bacterial community composition differs from that detected in other agro-ecosystems (Chen et al., 2014). Among these taxa, Chloroflexi, a representative oligotrophic taxa that favors nutrient-poor conditions, was less abundant in FU than in FL. In contrast, the relative abundance of copiotrophic taxa, including Proteobacteria, was higher in FU than in FL. Proteobacteria, especially Betaproteobacteria, are copiotrophic and favor nutrient-rich conditions with a high C content (Fierer et al., 2007; Newton and McMahon, 2011; Philippot et al., 2013; Wang et al., 2017). Unfortunately, we only analyzed the background surface soil, rather than soil samples collected from plant rows in the FU and FU treatments during the seedling period. Thus, the increased abundance of Proteobacteria in FU may be because of the increased supply of nutrient substrates that improved their growth. Bacteroidetes are copiotrophic and saprophytic bacteria (Fierer et al., 2007) that live in anaerobic environments (Xu et al., 2017). In our study, the abundance of Bacteroidetes was increased in FU. This may have been because of the higher SOM in FU than in FL. In addition, increased soil moisture favors the growth of Bacteroidetes (Xu et al., 2017).

### Effects of FU on Soil Properties in the Second Year

Compared with FL, FU reduced the soil salinity in plant belts in the second growth year spring, consistent with the results of a previous study (Dong et al., 2010). The salinity level was lower in furrows in FU than in seeding rows in FL. This is because salinity moves upwards along a capillary gradient in the spring when there is little rainfall, and accumulates on the surface of the soil ridges. A previous study showed that FU can lead to a heterogeneous distribution of salts in the plant root zone (Dong et al., 2010), and our previous study showed that alfalfa plants grow better with heterogeneous salinity than with uniform salinity in the root zone (Sun et al., 2016). The results of the present study show that FU alleviated salt stress in alfalfa in the second growth year.

In this study, the FU system significantly affected the soil physico-chemical properties and enhanced the contents of available nutrients in soil (Table 2), similar to the results of a previous study (Sun et al., 2017a). Our results showed that soil available K, total N, and SOM were significantly correlated with observed species and richness (Chao 1) indexes of soil bacterial communities. In our study, the observed species and Chao 1 index values were higher in FU than in FL. Previous studies have indicated that soil moisture content and other physicochemical properties affect microbial populations and community composition (Li and Sarah, 2003; Nguyen et al., 2018), which in turn enhances the formation and decomposition of organic matter (Nair and Ngouajio, 2012; Jing et al., 2013). In our study, the FU system enhanced the SOM, soil total N, and soil available K in the second growth year, possibly as a result of changes in soil microbial populations and community composition.

### Effects of FU on Alfalfa Yield and Quality

**Figure 10:**
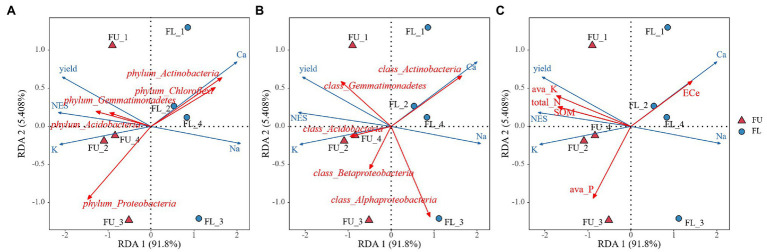
Redundancy analysis (RDA) diagram illustrating the relationship between the plant characteristics between soil bacterial community composition at phylum-level **(A)** class-level **(B)** and soil properties **(C)** from two seeding patterns.

The FU system increased alfalfa yield compared with the traditional FL system. The yield of alfalfa in the first cut of the second growth year was 37.4% higher in FU than in FL. Previous studies have also reported that FU systems can improve plant production (Li et al., 2001, 2013). In our study, Pearson’s correlation analyses showed that alfalfa yield was positively correlated with seedling emergence, soil available K, soil total N, SOM, and shoot K^+^ concentration, and negatively correlated with shoot Na^+^ concentration (Table 4), similarly result were found using RDA analysis (Figure 10), indicating that the increased yield was due to high emergence during the seedling stage, and greater nutrient availability for alfalfa uptake. Pearson’s correlation analyses showed that there was no significant relationship between soil ECe and yield (Table 4), however, RDA analysis shown soil ECe was negatively correlated with alfalfa yield (*p* < 0.05) (Figure 10C). In our experiment soil salinity was lower in FU than in FL, and that the alfalfa shoot Na^+^ concentration was lower in FU than in FL, suggesting that FU alleviated salinity and improved alfalfa production. Similar results were found in a previous study (Dong et al., 2010). During the whole plant growth period in our study, plant height was higher in FU than in FL in the second growth year spring, and this is an important yield component for alfalfa.

Notably, FU improved alfalfa quality compared with FL. For the first cut of alfalfa, the CP content was higher and NDF, ADF, and ash contents in alfalfa plants were lower in FU than in FL in the second year. The results of the Spearman correlation analyses revealed that the alfalfa CP content was positively correlated with soil total N and SOM (Figure 9). Thus, the increased alfalfa CP in FU was related to the increased soil total N and SOM in the FU system, while the decreased ash content in FU was due to higher soil available K, total N, and SOM. Another important aspect of the improved alfalfa quality in FU was the higher left-stem height, because the CP was lower and NDF, ADF, and ash contents were higher at the basal part of stem. In our experiment, we only compared the responses of alfalfa to the two seeding pattern at the first and second growth year. Response of alfalfa to FU pattern in the other growth year and over longer exposure to salinity soil need further study.

In conclusion, compared with FL, FU increased the soil moisture content and seedling emergence and altered the soil bacterial community during the seedling stage. The FU system also increased the available soil nutrient contents and decreased soil salinity at the returning green stage in the second growth year. Compared with FL, the FU system resulted in significant improvements in alfalfa plant height, yield, and quality, and decreased salt injury. The improved yield was due to higher soil moisture content, higher seedling emergence, higher contents of soil available nutrients and SOM, and lower salt content in the plant rows in spring of the second growth year. Enhanced K^+^ and reduced Na^+^ accumulation in shoots may be mechanisms to avoid salinity stress. Overall, our results show that specific cultural practices, such as FU, with sowing in early fall have immense potential for alleviating salt stress and improving alfalfa productivity and quality in saline fields. Additionally, FU with sowing in early fall with no need for mulching with plastic, it is inexpensive and do not pollute the soil. Therefore, FU is the effective way of sustaining the alfalfa productivity in saline soil.

## Data Availability Statement

The datasets presented in this study can be found in online repositories. The names of the repository/repositories and accession number(s) can be found at: https://www.ncbi.nlm.nih.gov/, PRJNA826619.

## Author Contributions

KJ and JS conceived and designed the research. JS, JZ, LY, and TZ conducted the experiment and collected the field data. JS and JZ analyzed the data. KJ and JS wrote the manuscript. All authors reviewed and edited the manuscript. All authors contributed to the article and approved the submitted version.

## Funding

This work was supported by Key Projects in Science and Technology of Inner Mongolia (2021ZD0031), Natural Science Foundation of Inner Mongolia (2018MS03028), Agricultural Science and Technology Innovation Program of CAAS (27-GRI-01), and Science and Technology Project from Inner Mongolia (2021GG0068 and 2019GG260).

## Conflict of Interest

The authors declare that the research was conducted in the absence of any commercial or financial relationships that could be construed as a potential conflict of interest.

## Publisher’s Note

All claims expressed in this article are solely those of the authors and do not necessarily represent those of their affiliated organizations, or those of the publisher, the editors and the reviewers. Any product that may be evaluated in this article, or claim that may be made by its manufacturer, is not guaranteed or endorsed by the publisher.

## Supplementary Material

Supplementary Figure S1 Relative abundance (% of individual taxonomic group) of dominant bacteria genera (mean ± SE, *n* = 4) in microbial communities following FU and FL. Different lowercase capital letters indicate statistically significant difference among communities at *p* = 0.05.

## References

[ref1] Allen S. G. Dobrenz A. K. Schonhorst M. H. Stoner J. E. (1985). Heritability of Nacl tolerance in germinating alfalfa seeds. Aoron. J. 77, 99–101. doi: 10.2134/agronj1985.00021962007700010023x

[ref2] Anower M. R. Mott I. W. Peel M. D. Wu Y. (2013). Characterization of physiological responses of two alfalfa half-sib families with improved salt tolerance. Plant Physiol. Biochem. 71, 103–111. doi: 10.1016/j.plaphy.2013.06.026, PMID: 23911728

[ref3] Ashraf M. Mcneilly T. Bradshaw A. D. (1987). Selection and heritability of tolerance to sodium-chloride in four forage species. Crop. Sci. 27, 232–234. doi: 10.2135/cropsci1987.0011183X002700020021x

[ref4] Bainard L. D. Hamel C. Gan Y. (2016). Edaphic properties override the influence of crops on the composition of the soil bacterial community in a semiarid agroecosystem. Appl. Soil Ecol. 105, 160–168. doi: 10.1016/j.apsoil.2016.03.013

[ref5] Bao S. D. (2018). Soil and Agricultural Chemistry Analysis. Beijing: China Agriculture Press.

[ref6] Battin T. J. Kaplan L. A. Newbold J. D. Hansen C. M. E. (2003). Contributions of microbial biofilms to ecosystem processes in stream mesocosms. Nature 426, 439–442. doi: 10.1038/nature02152, PMID: 14647381

[ref7] Chen Y. Wen X. Sun Y. Zhang J. Wu W. Liao Y. (2014). Mulching practices altered soil bacterial community structure and improved orchard productivity and apple quality after five growing seasons. Sci. Hortic. 172, 248–257. doi: 10.1016/j.scienta.2014.04.010

[ref8] Cheng Q. Chang H. Yang X. Wang D. Wang W. (2021). Salinity and nutrient modulate soil bacterial communities in the coastal wetland of the Yellow River Delta, China. Environ. Sci. Pollut. Res. 28, 14621–14631. doi: 10.1007/s11356-020-11626-x, PMID: 33219506

[ref9] Chi Z. Wang W. Li H. Wu H. Yan B. (2021). Soil organic matter and salinity as critical factors affecting the bacterial community and function of *Phragmites australis* dominated riparian and coastal wetlands. Sci. Total Environ. 762:143156. doi: 10.1016/j.scitotenv.2020.143156, PMID: 33131883

[ref10] Cuello J. P. Hwang H. Y. Gutierrez J. Kim S. Y. Kim P. J. (2015). Impact of plastic film mulching on increasing greenhouse gas emissions in temperate upland soil during maize cultivation. Appl. Soil Ecol. 91, 48–57. doi: 10.1016/j.apsoil.2015.02.007

[ref11] Deinlein U. Stephan A. B. Horie T. Luo W. Xu G. Schroeder J. I. (2014). Plant salt-tolerance mechanisms. Trends Plant Sci. 19, 371–379. doi: 10.1016/j.tplants.2014.02.001, PMID: 24630845 PMC4041829

[ref12] Devkota M. Martius C. Gupta R. K. Devkota K. P. Mcdonald A. J. Lamers J. P. A. (2015). Managing soil salinity with permanent bed planting in irrigated production systems in Central Asia. Agric. Ecosyst. Environ. 202, 90–97. doi: 10.1016/j.agee.2014.12.006

[ref13] Dong H. Kong X. Luo Z. Li W. Xin C. (2010). Unequal salt distribution in the root zone increases growth and yield of cotton. Eur. J. Agron. 33, 285–292. doi: 10.1016/j.eja.2010.08.002

[ref14] Dong H. Li W. Tang W. Zhang D. (2009). Early plastic mulching increases stand establishment and lint yield of cotton in saline fields. Field Crop Res 111, 269–275. doi: 10.1016/j.fcr.2009.01.001

[ref15] Esechie H. A. Al-Barhi B. Al-Gheity S. Al-Khanjari S. (2002). Root and shoot growth in salinity-stressed alfalfa in response to nitrogen source. J. Plant Nutr. 25, 2559–2569. doi: 10.1081/PLN-120014713

[ref16] Fan Z. L. Ma Y. J. Ma Y. J. (2001). Salinized soils and their improvement and utilization in West China. Arid Zone Res. 18, 1–6. doi: 10.13866/j.azr.2001.03.001

[ref17] Fierer N. Bradford M. A. Jackson R. B. (2007). Toward an ecological classification of soil bacteria. Ecology 88, 1354–1364. doi: 10.1890/05-1839 17601128

[ref18] Gao Y. Wang J. Guo S. Hu Y. Li T. Mao R.. (2015). Effects of salinization and crude oil contamination on soil bacterial community structure in the Yellow River Delta region, China. Appl. Soil Ecol. 86, 165–173. doi: 10.1016/j.apsoil.2014.10.011

[ref19] Gu Y. J. Han C. L. Kong M. Shi X. Y. Zdruli P. Li F. M. (2018). Plastic film mulch promotes high alfalfa production with phosphorus-saving and low risk of soil nitrogen loss. Field Crop Res 229, 44–54. doi: 10.1016/j.fcr.2018.09.011

[ref20] Guo X. P. Lu D. P. Niu Z. S. Feng J. N. Chen Y. R. Tou F. Y.. (2018). Bacterial community structure in response to environmental impacts in the intertidal sediments along the Yangtze estuary, China. Mar. Pollut. Bull. 126, 141–149. doi: 10.1016/j.marpolbul.2017.11.003, PMID: 29421081

[ref21] Jing T. Lu S. Fan M. Li X. Kuzyakov Y. (2013). Labile soil organic matter fractions as influenced by non-flooded mulching cultivation and cropping season in rice-wheat rotation. Eur. J. Soil Biol. 56, 19–25. doi: 10.1016/j.ejsobi.2013.02.001

[ref22] Jurburg S. D. Natal-da-Luz T. Raimundo J. Morais P. V. Sousa J. P. van Elsas J. D.. (2018). Bacterial communities in soil become sensitive to drought under intensive grazing. Sci. Total Environ. 618, 1638–1646. doi: 10.1016/j.scitotenv.2017.10.012, PMID: 29054674

[ref23] Kargas G. Kerkides P. Seyfried M. Sgoumbopoulou A. (2011). WET sensor performance in organic and inorganic media with heterogeneous moisture distribution. Soil Sci. Soc. Am. J. 75, 1244–1252. doi: 10.2136/sssaj2010.0238

[ref24] Krishnamoorthy U. Muscato T. V. Sniffen C. J. Van Soest P. J. (1982). Nitrogen fractions in selected feedstuffs. J. Dairy Sci. 65, 217–225. doi: 10.3168/jds.S0022-0302(82)82180-2

[ref25] Kunzova E. Hejcman M. (2009). Yield development of winter wheat over 50 years of FYM, N, P and K fertilizer application on black earth soil in the Czech Republic. Field Crop Res 111, 226–234. doi: 10.1016/j.fcr.2008.12.008

[ref26] Latrach L. Farissi M. Mouradi M. Makoudi B. Bouizgaren A. Ghoulam C. (2014). Growth and nodulation of alfalfa-rhizobia symbiosis under salinity: electrolyte leakage, stomatal conductance, and chlorophyll fluorescence. Turk. J. Agric. For. 38, 320–326. doi: 10.3906/tar-1305-52

[ref27] Li X. Y. Gong J. D. Gao Q. Z. Li F. R. (2001). Incorporation of ridge and furrow method of rainfall harvesting with mulching for crop production under semiarid conditions. Agric Water Manag 50, 173–183. doi: 10.1016/S0378-3774(01)00105-6

[ref28] Li X. Y. Gong J. D. Wei X. H. (2000). In-situ rainwater harvesting and gravel mulch combination for corn production in the dry semi-arid region of China. J. Arid Environ. 46, 371–382. doi: 10.1006/jare.2000.0705

[ref29] Li R. Hou X. Jia Z. Han Q. Ren X. Yang B. (2013). Effects on soil temperature, moisture, and maize yield of cultivation with ridge and furrow mulching in the rainfed area of the loess plateau, China. Agric Water Manag 116, 101–109. doi: 10.1016/j.agwat.2012.10.001

[ref30] Li Y. Kang Z. Song B. Wang J. Zhang X. Wang J.. (2021). Soil salinity and nutrients availability drive patterns in bacterial community and diversity along succession gradient in the Yellow River Delta. Estuar. Coast. Shelf Sci. 262:107621. doi: 10.1016/j.ecss.2021.107621

[ref31] Li X. Sarah P. (2003). Enzyme activities along a climatic transect in the Judean Desert. Catena 53, 349–363. doi: 10.1016/S0341-8162(03)00087-0

[ref32] Li X. Su D. Yuan Q. (2007). Ridge-furrow planting of alfalfa (*Medicago sativa* L.) for improved rainwater harvest in rainfed semiarid areas in Northwest China. Soil Tillage Res. 93, 117–125. doi: 10.1016/j.still.2006.03.022

[ref33] Liao Y. Cao H. X. Xue W. K. Liu X. (2021). Effects of the combination of mulching and deficit irrigation on the soil water and heat, growth and productivity of apples. Agric Water Manag 243:106482. doi: 10.1016/j.agwat.2020.106482

[ref304] Liao R. Wu Y. Hu Y. Xu D. Huang Q. Wang S. (2019). Micro-irrigation strategies to improve water-use of cherry trees in Northern China. Agric. Water Manage. 221, 388–396. doi: 10.1016/j.agwat.2019.05.017

[ref34] Ling N. Chen D. Guo H. Wei J. Bai Y. Shen Q.. (2017). Differential responses of soil bacterial communities to long-term N and P inputs in a semi-arid steppe. Geoderma 292, 25–33. doi: 10.1016/j.geoderma.2017.01.013

[ref35] Liu Y. Li S. Chen F. Yang S. Chen X. (2010a). Soil water dynamics and water use efficiency in spring maize (*Zea mays* L.) fields subjected to different water management practices on the loess plateau. China Agric. Water Manag. 97, 769–775. doi: 10.1016/j.agwat.2010.01.010

[ref36] Liu X. E. Li X. G. Hai L. Wang Y. P. Li F. M. (2014). How efficient is film fully-mulched ridge-furrow cropping to conserve rainfall in soil at a rainfed site? Field Crop Res 169, 107–115. doi: 10.1016/j.fcr.2014.09.014

[ref37] Liu Y. Yang S. Li S. Chen X. Chen F. (2010b). Growth and development of maize (*Zea mays* L.) in response to different field water management practices: resource capture and use efficiency. Agric. For. Meteorol. 150, 606–613. doi: 10.1016/j.agrformet.2010.02.003

[ref38] Logue J. Stedmon C. Kellerman A. Nielsen N. Andersson A. Laudon H.. (2016). Experimental insights into the importance of aquatic bacterial community composition to the degradation of dissolved organic matter. ISME J. 10, 533–545. doi: 10.1038/ismej.2015.131, PMID: 26296065 PMC4817675

[ref39] Meiri A. Plaut Z. (1985). Crop production and management under saline conditions. Plant and Soil 89, 253–271. doi: 10.1007/bf02182246

[ref40] Munns R. Tester M. (2008). Mechanisms of salinity tolerance. Annu. Rev. Plant Biol. 59, 651–681. doi: 10.1146/annurev.arplant.59.032607.092911 18444910

[ref41] Nair A. Ngouajio M. (2012). Soil microbial biomass, functional microbial diversity, and nematode community structure as affected by cover crops and compost in an organic vegetable production system. Appl. Soil Ecol. 58, 45–55. doi: 10.1016/j.apsoil.2012.03.008

[ref42] Nakamura A. Tun C. C. Asakawa S. Kimura M. (2003). Microbial community responsible for the decomposition of rice straw in a paddy field: estimation by phospholipid fatty acid analysis. Biol. Fertil. Soils 38, 288–295. doi: 10.1007/s00374-003-0658-6

[ref43] Newton R. J. McMahon K. D. (2011). Seasonal differences in bacterial community composition following nutrient additions in a eutrophic lake. Environ. Microbiol. 13, 887–899. doi: 10.1111/j.1462-2920.2010.02387.x, PMID: 21138514

[ref44] Nguyen L. T. T. Osanai Y. Lai K. Anderson I. C. Bange M. P. Tissue D. T.. (2018). Responses of the soil microbial community to nitrogen fertilizer regimes and historical exposure to extreme weather events: flooding or prolonged-drought. Soil Biol. Biochem. 118, 227–236. doi: 10.1016/j.soilbio.2017.12.016

[ref45] Nie Y. Wang M. Zhang W. Ni Z. Hashidoko Y. Shen W. (2018). Ammonium nitrogen content is a dominant predictor of bacterial community composition in an acidic forest soil with exogenous nitrogen enrichment. Sci. Total Environ. 624, 407–415. doi: 10.1016/j.scitotenv.2017.12.142, PMID: 29262382

[ref46] Noble C. L. Halloran G. M. West D. W. (1984). Identification and selection for salt tolerance in lucerne (*Medicago sativa* L.). Aust. J. Agr. Res. 35, 239–252. doi: 10.1071/ar9840239

[ref47] Philippot L. Raaijmakers J. M. Lemanceau P. van der Putten W. H. (2013). Going back to the roots: the microbial ecology of the rhizosphere. Nat. Rev. Microbiol. 11, 789–799. doi: 10.1038/nrmicro3109, PMID: 24056930

[ref48] Robertson J. B. Van Soest P. J. (1981). “The detergent system of analysis and its application to human foods,” in The Analysis of Dietary Fiber in Food. eds. James W. P. T. Theander O. (New York, NY, USA: Marcel Dekker), 123–158.

[ref50] Silvente S. Sobolev A. P. Lara M. (2012). Metabolite adjustments in drought tolerant and sensitive soybean genotypes in response to water stress. PLoS One 7:e38554. doi: 10.1371/journal.pone.0038554, PMID: 22685583 PMC3369847

[ref51] Summers CG Putna DH. (2008). Irrigated Alfalfa Management for Mediterranean and Desert Zones. University of California Agriculture and Natural Resources, California

[ref52] Sun C. T. Feng D. Mi Z. R. Li C. X. Zhang J. P. Gao Y.. (2017a). Impacts of ridge-furrow planting on salt stress and cotton yield under drip irrigation. Watermark 9:49. doi: 10.3390/w9010049

[ref53] Sun J. Yang G. Zhang W. Zhang Y. (2016). Effects of heterogeneous salinity on growth, water uptake, and tissue ion concentrations of alfalfa. Plant and Soil 408, 211–226. doi: 10.1007/s11104-016-2922-1

[ref54] Sun J. Yu L. Zhao J. Liu H. Zhang Y. (2017b). Effects of heterogeneous root zone salinity on plant growth and ion characteristic in alfalfa. Sci. Agric. Sin. 50, 4329–4306. doi: 10.3864/j.issn.0578-1752.2017.22.006

[ref55] Suo G. D. Xie Y. S. Zhang Y. Luo H. (2019). Long-term effects of different surface mulching techniques on soil water and fruit yield in an apple orchard on the loess plateau of China. Sci. Hortic. 246, 643–651. doi: 10.1016/j.scienta.2018.11.028

[ref56] Van Soest P. J. Robertson J. B. Lewis B. A. (1991). Methods for dietary fiber, neutral detergent fiber, and nonstarch polysaccharides in relation to animal nutrition. J. Dairy Sci. 74, 3583–3597. doi: 10.3168/jds.S0022-0302(91)78551-2, PMID: 1660498

[ref57] Wakelin S. A. Colloff M. J. Harvey P. R. Marschner P. Gregg A. L. Rogers S. L. (2007). The effects of stubble retention and nitrogen application on soil microbial community structure and functional gene abundance under irrigated maize. FEMS Microbiol. Ecol. 59, 661–670. doi: 10.1111/j.1574-6941.2006.00235.x, PMID: 17116166

[ref58] Wang J. Song Y. Ma T. Raza W. Li J. Howland J. G.. (2017). Impacts of inorganic and organic fertilization treatments on bacterial and fungal communities in a paddy soil. Appl. Soil Ecol. 112, 42–50. doi: 10.1016/j.apsoil.2017.01.005

[ref59] Xiong X. Wei Y.-q. Chen J.-h. Liu N. Zhang Y.-j. (2020). Transcriptome analysis of genes and pathways associated with salt tolerance in alfalfa under non-uniform salt stress. Plant Physiol. Biochem. 151, 323–333. doi: 10.1016/j.plaphy.2020.03.035, PMID: 32251957

[ref60] Xu S. Lu W. Liu Y. Ming Z. Liu Y. Meng R.. (2017). Structure and diversity of bacterial communities in two large sanitary landfills in China as revealed by high-throughput sequencing (MiSeq). Waste Manag. 63, 41–48. doi: 10.1016/j.wasman.2016.07.047, PMID: 27515184

[ref61] Yang Y. Dou Y. An S. (2018). Testing association between soil bacterial diversity and soil carbon storage on the loess plateau. Sci. Total Environ. 626, 48–58. doi: 10.1016/j.scitotenv.2018.01.081, PMID: 29335174

[ref62] Yang W. Jeelani N. Zhu Z. H. Luo Y. Q. Cheng X. L. An S. Q. (2019). Alterations in soil bacterial community in relation to *Spartina alterniflora* Loisel. Invasion chronosequence in the eastern Chinese coastal wetlands. Appl. Soil Ecol. 135, 38–43. doi: 10.1016/j.apsoil.2018.11.009

[ref305] Zhang S. Li P. Yang X. Wang Z. Chen X. (2011). Effects of tillage and plastic mulch on soil water, growth and yield of spring-sown maize. Soil Tillage Res. 112, 92–97. doi: 10.1016/j.still.2010.11.006

[ref63] Zhao Q. Bai J. Gao Y. Zhao H. Zhang G. Cui B. (2020). Shifts in the soil bacterial community along a salinity gradient in the Yellow River Delta. Land Degrad. Dev. 31, 2255–2267. doi: 10.1002/ldr.3594

[ref64] Zribi W. Aragues R. Medina E. Faci J. M. (2015). Efficiency of inorganic and organic mulching materials for soil evaporation control. Soil Tillage Res. 148, 40–45. doi: 10.1016/j.still.2014.12.003

