# Supplementary figures and images for: Effects of a Furrow-Bed Seeding System on Stand Establishment, Soil Bacterial Diversity, and the Yield and Quality of Alfalfa Under Saline Condition
Source: Front Plant Sci. 2022 Jun 9;13:919912. doi: 10.3389/fpls.2022.919912 (PMC9225151; doi:10.3389/fpls.2022.919912)

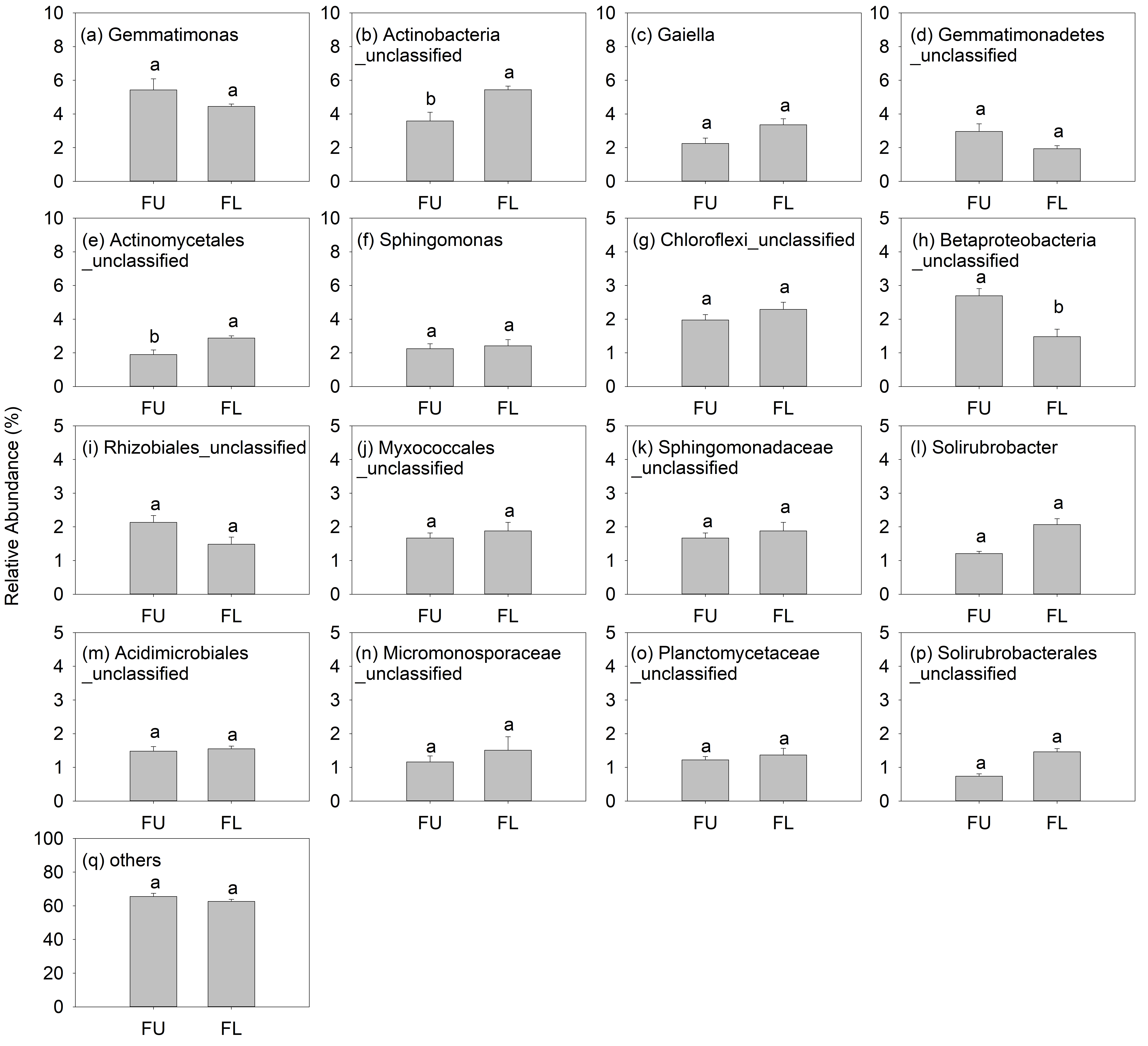

Supplement: Supplementary Figure S1 — Relative abundance (% of individual taxonomic group) of dominant bacteria genera (mean ± SE, n = 4) in microbial communities following FU and FL. Different lowercase capital letters indicate statistically significant difference among communities at p = 0.05. [file Image_1.JPEG]
